# Plasma metabolomic analysis of human hepatocellular carcinoma: Diagnostic and therapeutic study

**DOI:** 10.18632/oncotarget.10119

**Published:** 2016-06-17

**Authors:** Yang Chen, Jianyin Zhou, Jinquan Li, Jianghua Feng, Zhong Chen, Xiaomin Wang

**Affiliations:** ^1^ Department of Electronic Science, Fujian Provincial Key Laboratory of Plasma and Magnetic Resonance, Xiamen University, Xiamen, 361005, China; ^2^ Department of Hepatobiliary and Pancreatic Surgery, Zhongshan Hospital, Xiamen University, Xiamen, 361004, China

**Keywords:** hepatocellular carcinoma, metabolic profiling, ^1^H-NMR spectroscopy, transcatheter arterial chemoembolization, surgery

## Abstract

Many hepatocellular carcinoma (HCC) patients suffer from late stages when diagnosed, leading to dismal prospects for cure. Improving the diagnosis and treatment of HCC remains a challenge. In this work, NMR-based metabolomic techniques were used to investigate the metabolic alterations of HCC patients from different pathological backgrounds. Metabolic improvement of clinical surgical treatments or transcatheter arterial chemoembolization (TACE) for recurrent or metastatic HCC was also evaluated. HCC was characterized by enhanced lipid metabolism and high consumption in response to liver injury. Expectedly, higher consumption of glucose and lactate production in TACE group confirmed that recurrent or metastatic HCC is more active in citric acid cycle and oxidative phosphorylation. However, TACE or surgical treatments did not immediately improve the metabolic profiles of HCC patients. Combining multivariate statistical analyses with univariate *t*-test, a series of characteristic metabolites were identified and served as biomarkers for discrimination of HCC patients in different pathological backgrounds. The relative metabolic pathway analyses help to get insight into the underlying biochemical mechanism and extend clinical relevance. Furthermore, algorithm of support vector classification was used to identify HCC and control subjects, and diagnostic sensitivity and specificity reached to 100% and 81.08% respectively by receiver operating characteristic analysis. It is concluded that NMR-based metabolomic analysis of plasma can provide a powerful approach to discover diagnostic and therapeutic biomarkers, and subsequently contribute to clinical disease management.

## INTRODUCTION

Human hepatocellular carcinoma (HCC), with the third highest mortality, is one of the most common malignant tumors in the world. Most deaths from liver diseases are attributed to HCC [1]. Even though more than half of the cases come from China, a dramatically increasing incidence of HCC in the world has recently been reported in developed countries such as France, Japan, the UK, and the USA [2]. As an aggressive tumor, HCC is largely occurring on individuals with a previous liver disease. Although it can be clinically detectable by tissue-based histopathological evaluation and/or blood-based biochemical assays, notably by expression of alpha-fetoprotein (AFP) in the blood of advanced cases. However, these standard approaches may be unqualified and suffer from a lack of both sensitivity and specificity for early diagnosis [3]. Many patients have had advanced HCC stages when diagnosed and the prospects for cure are dismal. Consequently, there is an urgent need to seek new biomarkers for accurate diagnosis of HCC.

Recently, metabolomics has been proved to be a highly successful approach that is capable of detecting metabolic changes under different pathophysiological status [4]. By measuring changes in metabolite concentrations in the biological tissues or biofluids, the mutagenicity and progression of a disease can be determined and monitored [5, 6]. Metabolomics based on nuclear magnetic resonance (NMR) spectroscopy or mass spectroscopy could be used to identify biomarkers for specific pathological or physiological status. Metabolomic analysis of human tissues and biofluids has been increasingly used to unveil metabolic alterations associated with different cancer types, such as breast [7], kidney [8], lung [9], prostate [10], and colorectal cancers [11]. In the case of liver diseases, a number of studies have been focusing on the metabolic profiling of tissues or biofluids. Shariff *et al.* and Wu *et al.* respectively reported that a set of urinary metabolites (creatinine, carnitine, creatine, and acetone, *etc*) can be used to identify metabolic changes associated with HCC [12, 13] in Nigerian and Chinese populations. Apolito and colleagues performed tissue-based liquid chromatography-mass spectrometry to discriminate primary HCC from hepatic colorectal metastases via the changes of basic amino acids (arginine, citrulline, ornithine) [14]. Differential metabolites including alanine, leucine, and glucose have been identified to define hepatic tumorigenesis [15, 16]. Moreover, metabolic abnormalities have also been demonstrated by serum and plasma studies associated with the severity of liver disease. Fages *et al*. found sixteen metabolites in serum were significantly associated with HCC risk of a European prospective cohort [17]. Other studies show significant differences between compensated and decompensated cirrhosis, and between alcoholic cirrhosis and viral hepatitis [18–20]. The influence of hepatitis infection and potential liver damage were simultaneously assessed [21, 22]. To date, several NMR or mass spectrometry-based serum metabolomic studies have been conducted to study HCC [17, 19, 20, 23]. And discriminatory metabolic alterations in HCC patients could draw a basic conclusion that altered mitochondrial respiration and glycolytic pathways lead to altered metabolic profiles in tumor cells [24, 25], but the identified metabolites are not concordant across these studies. Hence, these identified metabolites need to be further validated, not only that, global metabolic evaluation need to be simultaneously explored and studied to see alterations in the clinical treatment response or in the prognoses associated with recurrence or metastasis of HCC.

In this work, we present plasma metabolic profiling via ^1^H NMR spectra of HCC patients from the different pathological backgrounds in comparison with healthy humans. Our aims are: (i) to depict the plasma metabolic characteristics of HCC patients for validation purpose; and (ii) to investigate potential metabolic alterations of postsurgical treatment and prognoses.

## RESULTS AND DISCUSSION

### Discrimination of metabolic profiles between control and HCC patients

**Figure 1:**
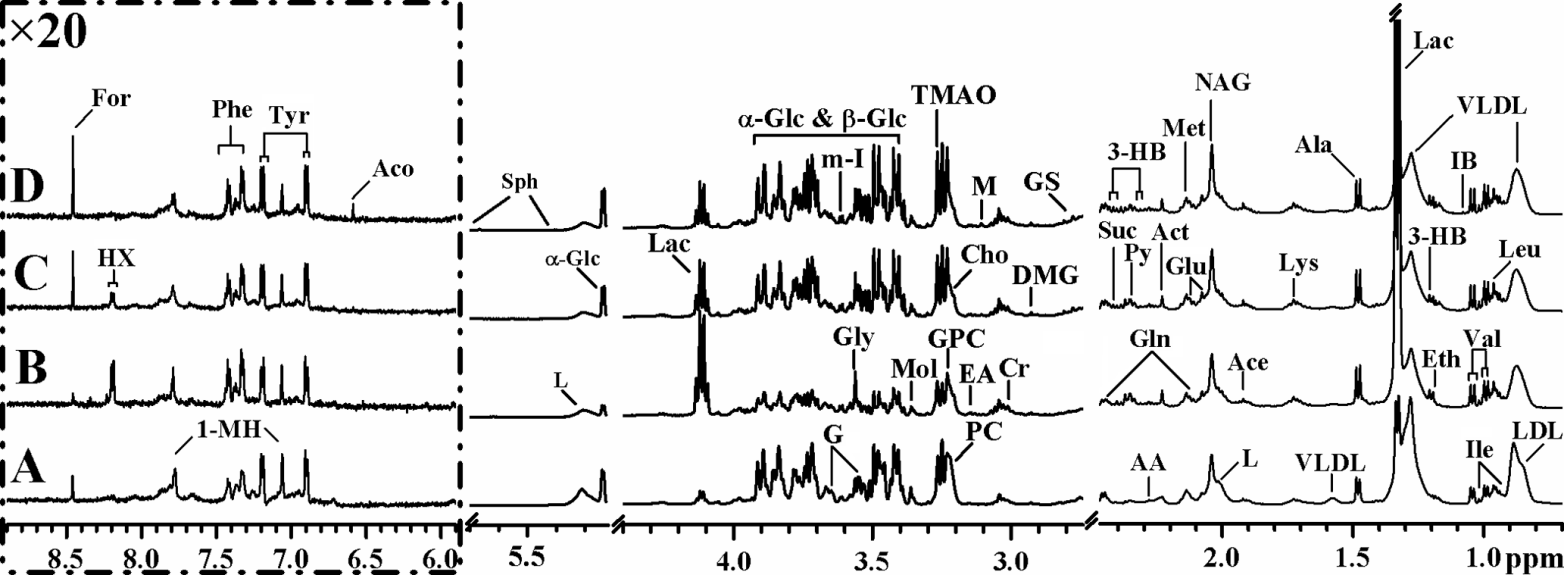
Average ^1^H NMR spectra of plasma samples from different pathological backgrounds (**A**) Healthy control; (**B**) TACE for recurrence or metastasis of HCC; (**C**) HCC only subject; (**D**) post-operative HCC subject. Keys for the assignments are shown in Table 2 and Table S1.

A total of 168 spectra of plasma samples were acquired from different groups, including healthy controls (*n* = 60), HCC only subjects (*n* = 24), transcatheter arterial chemoembolization (TACE) for recurrence or metastasis of HCC subjects (*n* = 18), and surgery related HCC subjects (*n* = 33 for pre- and post-operative, respectively). The average NMR spectra of plasma samples from different pathological backgrounds are shown in Figure 1, and the resonance assignments are summarized in Table S1 in Supplemental Information. Plasma contains all of the low molecular weight metabolites including glucose, amino acids, organic acids, and metabolic intermediates and end-products. Some high molecular weight metabolites such as lipoproteins, fatty acids and phospholipids were also observed. NMR spectra of plasma under similar physiological conditions are highly reproducible, thus making them helpful for the diagnosis of diseased states.

Inspection of the ^1^H NMR spectra reveals some obvious metabolic changes in HCC patients (with- or without- treatments) compared to healthy controls. For example, significantly decreased LDL, VLDL, and lipid concentrations and increased acetate and lactate concentrations are observed, which is consistent with previous reports of both rat model and human HCC patients [19, 26]. Such observation is possibly due to enhanced lipid metabolism in response to liver injury caused by HCC. The increased levels of alanine, lactate, and pyruvate in intermediates of glycolysis and TCA cycle suggest high consumption of glucose by HCC in response to the stimulated aerobic glycolysis or the Warburg effect with conversion through pyruvate to alanine and lactate [27, 28]. Notably, higher consumption of glucose and lactate production in TACE group than any other groups implied that recurrence or metastasis HCC is more active in conversion of glucose to lactic acid during mitochondrial citric acid cycle and oxidative phosphorylation. Besides, the levels of several free amino acids such as isoleucine, leucine, tyrosine, and valine have been shown to vary with different HCC groups. However, the more precise and detailed information need to be confirmed by multivariate analysis.

### Characteristic metabolites for HCC patients

**Figure 2:**
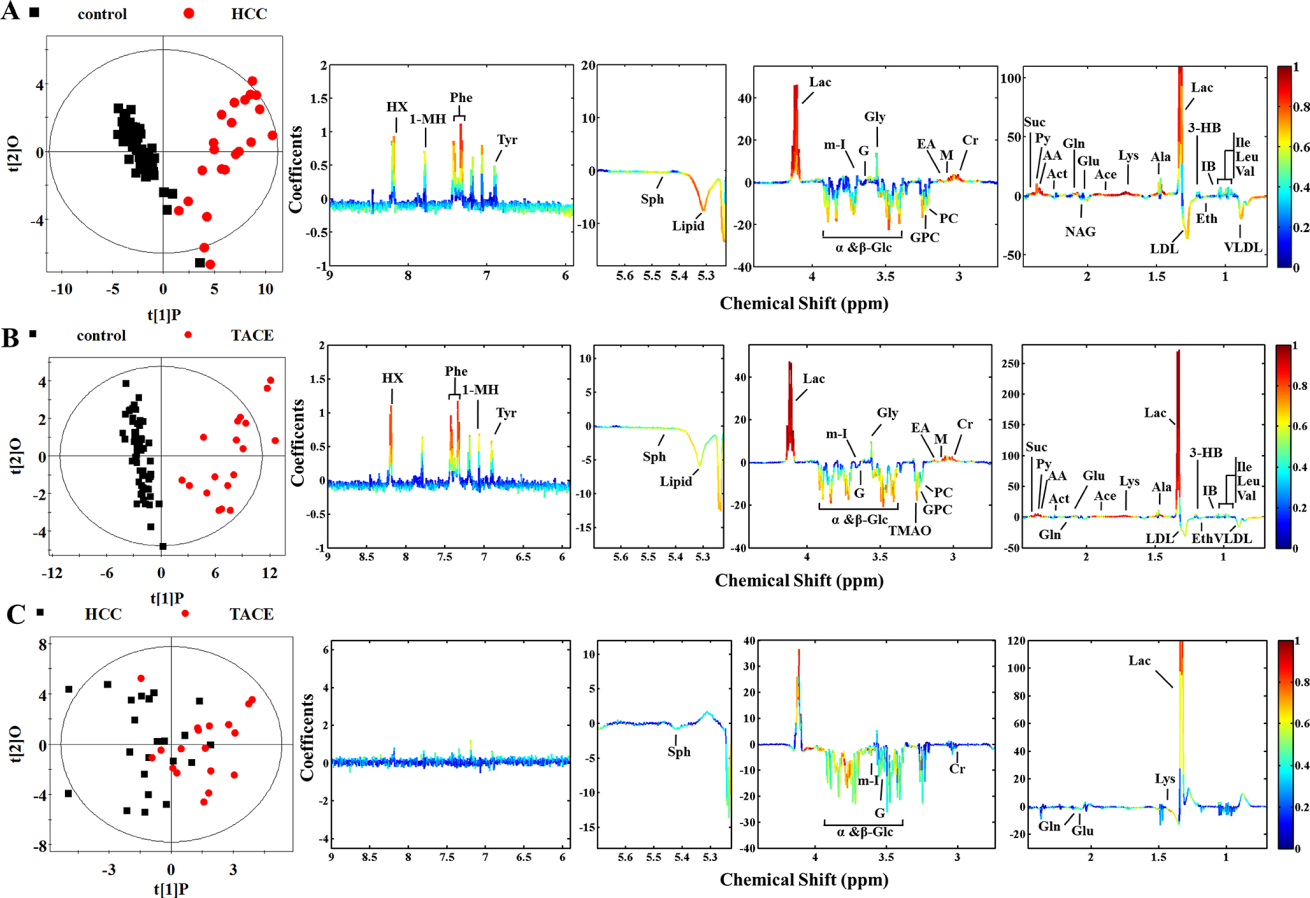
Scores and loading plots for determination of plasma metabolites responsible for the metabolic variations in different pair-wise pathological subjects via OPLS-DA analyses (**A**) Control versus HCC groups (R^2^X = 52.2%, R^2^Y = 0.836, Q^2^ = 0.798, *P* < 0.0001); (**B**) control versus TACE groups (R^2^X = 58.5%, R^2^Y = 0.880, Q^2^ = 0.849, *P* < 0.0001); (**C**) HCC versus TACE groups (R^2^X = 48.1%, R^2^Y = 0.381, Q^2^ = −0.041, *P* = 1).

Principal component analysis (PCA) was performed on the plasma ^1^H NMR data from different pair-wise groups to reveal trends and show clusters among the subjects. The PCA scores plots revealed an obvious separation between control (*n* = 60) and HCC patients (*n* = 24, without surgery or treatment) (Figure S1A), which implied abnormal metabolic pattern from HCC patients. Orthogonal projection to latent structure with discriminant analysis (OPLS-DA) was subsequently utilized to identify differential metabolites responsible for metabolic differences (Figure 2A). The reliability of OPLS-DA models could be evaluated by Q^2^ value and significant level of *P* value. Usually larger Q^2^ value described better reliability. To determine statistical significance for these selected metabolites, relative concentrations were compared by Student's *t*-test analysis. As a result, seven selected metabolites were considered not significant and excluded in the final list of characteristic metabolites (Table 1). Lower levels of glucose, glycerophosphocholine, LDL, lipid, sphingosine, and VLDL, together with higher levels of lactate, and certain amino acids such as alanine, lysine, and valine were observed in HCC group than that of control, revealing high consumption of glucose in HCC subjects. Our results share some characteristic metabolites with some similar studies [17, 19, 25], including acetate, ethanol, glucose, *etc*. Meanwhile, some novel characteristic metabolites in the present work, such as alanine, hypoxanthine, sphingosine, *etc*., were also identified. It is suggestive that some kinds of population-related metabolic alteration are associated with HCC state, but a series of biological behaviors caused by high glucose consumption have become a common feature.

**Table 1:** The relative concentrations of the characteristic metabolites in the plasma of HCC patients from different pathological backgrounds

Metabolite	Abbr.	Control	HCC	TACE	Pre-surgery	Post-surgery
low-density lipoprotein	LDL	13.91 ± 3.21*	9.51 ± 1.93^a^	9.82 ± 2.40^a^	9.74 ± 2.77	10.26 ± 2.22^a^
very low-density lipoprotein	VLDL	9.94 ± 4.04	6.58 ± 1.90^a^	6.45 ± 2.17^a^	6.64 ± 2.85	6.40 ± 2.22^a^
Isoleucine	Ile	1.03 ± 0.13	1.20 ± 0.38^a^	1.14 ± 0.17	1.19 ± 0.20	1.20 ± 0.14
Leucine	Leu	1.15 ± 0.16	1.44 ± 0.62^a^	1.36 ± 0.22	1.29 ± 0.29	1.24 ± 0.19^c^
Isobutyrate	IB	0.05 ± 0.02	0.08 ± 0.05^a^	0.08 ± 0.02	0.06 ± 0.04	0.06 ± 0.03^a^, ^b^
Ethanol	Eth	0.42 ± 0.23	0.27 ± 0.09^a^	0.23 ± 0.08^a^	0.66 ± 0.39	0.60 ± 0.31^a^, ^b^
3-Hydroxybutyrate	3-HB	0.99 ± 0.16	1.76 ± 0.61^a^	1.72 ± 0.50^a^	1.57 ± 0.65	1.57 ± 0.86 ^a^
Lactate	Lac	7.90 ± 1.57	25.19 ± 10.38^a^	30.82 ± 10.36^a^	10.32 ± 1.77	11.60 ± 3.09^a^, ^b^, ^c^
Alanine	Ala	1.13 ± 0.20	1.73 ± 0.60^a^	1.63 ± 0.36^a^	1.21 ± 0.39	1.12 ± 0.28^a^
Acetate	Ace	0.16 ± 0.05	0.25 ± 0.04	0.24 ± 0.05	0.25 ± 0.08	0.24 ± 0.08^a^
Lipid	Lipid	5.64 ± 1.64	3.93 ± 0.73^a^	3.96 ± 0.90^a^	4.21 ± 1.13	4.50 ± 1.01^a^
Glutamate	Glu	2.93 ± 0.45	3.67 ± 0.40	3.29 ± 0.47	4.01 ± 0.87	3.74 ± 0.65^a^
Acetone	Act	0.22 ± 0.09	0.29 ± 0.14^a^	0.33 ± 0.19^a^	0.27 ± 0.15	0.26 ± 0.20^a^
Acetoacetate	AA	0.10 ± 0.02	0.13 ± 0.03	0.12 ± 0.03	0.14 ± 0.06	0.13 ± 0.06^a^, ^b^
Pyruvate	Py	0.09 ± 0.03	0.43 ± 0.32^a^	0.33 ± 0.16^a^	0.19 ± 0.12	0.17 ± 0.08^a^, ^b^
Succinate	Suc	0.05 ± 0.02	0.14 ± 0.06^a^	0.14 ± 0.05^a^	0.11 ± 0.10	0.11 ± 0.09^a^
Guanidinosuccinate	GS	0.63 ± 0.12	0.68 ± 0.19^a^	0.67 ± 0.17	0.98 ± 0.47	1.05 ± 0.53^a^, ^b^
N,N-Dimethylglycine	DMG	0.06 ± 0.03	0.08 ± 0.02	0.08 ± 0.02	0.11 ± 0.07	0.12 ± 0.08^a^, ^b^
Creatine	Cr	0.53 ± 0.13	0.75 ± 0.15	0.70 ± 0.15	0.78 ± 0.19	0.77 ± 0.14^c^
Malonate	M	0.05 ± 0.03	0.08 ± 0.02	0.08 ± 0.02	0.13 ± 0.10	0.16 ± 0.14^a^, ^b^, ^c^
Ethanolamine	EA	0.12 ± 0.06	0.19 ± 0.05	0.21 ± 0.05	0.19 ± 0.13	0.21 ± 0.16^a^, ^b^
Phosphocholine	PC	0.67 ± 0.19	0.54 ± 0.14	0.52 ± 0.14	0.55 ± 0.16	0.51 ± 0.14^a^
β-Glucose	β-Glc	16.35 ± 3.58	9.57 ± 6.06^a^	7.29 ± 4.30	17.81 ± 3.36	17.10 ± 2.38^a^, ^b^
Trimethylamine N-oxide	TMAO	0.62 ± 0.15	0.56 ± 0.20	0.48 ± 0.18	0.91 ± 0.24	0.92 ± 0.23^a^
Methanol	Mol	0.39 ± 0.34	0.19 ± 0.05^a^	0.21 ± 0.10^a^	0.31 ± 0.15	0.25 ± 0.12^a^
α-Glucose	α-Glc	11.30 ± 2.48	6.49 ± 3.90^a^	4.96 ± 2.70	12.23 ± 2.21	11.61 ± 1.40^a^, ^b^
Glycerol	G	2.63 ± 1.00	2.41 ± 0.56^a^	2.22 ± 0.30^a^	2.85 ± 0.73	2.74 ± 0.38^a^
Sphingosine	Sph	0.09 ± 0.06	0.21 ± 0.02^a^	0.08 ± 0.04^a^	0.59 ± 0.06	0.18 ± 0.08^a^, ^b^
*cis*-Aconitate	Aco	0.01 ± 0.00	0.01 ± 0.01	0.01 ± 0.00^a^	0.03 ± 0.02	0.04 ± 0.01^a^, ^b^
Tyrosine	Tyr	0.14 ± 0.04	0.18 ± 0.06^a^	0.21 ± 0.07^a^	0.16 ± 0.09	0.19 ± 0.08^a^
1-Methylhistidine	1-MH	0.11 ± 0.05	0.13 ± 0.03^a^	0.14 ± 0.04	0.09 ± 0.09	0.10 ± 0.07
Phenylalanine	Phe	0.13 ± 0.05	0.33 ± 0.11^a^	0.36 ± 0.12^a^	0.19 ± 0.17	0.25 ± 0.14 ^a^
Hypoxanthine	HX	0.01 ± 0.00	0.09 ± 0.07^a^	0.11 ± 0.06^a^	0.05 ± 0.02	0.04 ± 0.02^a^, ^b^
Formate	For	0.04 ± 0.01	0.01 ± 0.00	0.01 ± 0.00	0.09 ± 0.05	0.10 ± 0.05^a^, ^b^

* The relative concentrations of metabolites are presented as mean ± SD of the integration value of the characteristic resonance of each metabolite.

Characteristic metabolite with *P* < 0.05 versus:

a control group,

b TACE, and

c preoperative subjects.

### HCC patients' metabolic responses to TACE and surgical treatments

To further investigate metabolic improvement of clinical treatment and prognoses associated with recurrence or metastasis of HCC, those patients who underwent TACE and surgical treatment were compared with control, respectively. As an intervention tool, treatment of TACE is often used in recurrent or metastatic liver cancer. Clearly, the TACE and control groups were separated into two classes along the first component on the PCA scores plots (Figure S1A in the Supplemental Information). The individual differences of TACE group seemed to be more significant than those of control group, suggesting different HCC states associated with recurrence or metastasis. However, the metabolic differences were still obvious (Figure 2B), implying unrecovered biological states of the TACE subjects. With regard to characteristic metabolites, higher levels of lactate, phenylalanine, and hypoxanthine and lower levels of glucose in TACE group gave the primary contribution to metabolic variations. Also, some other metabolites, including elevated levels of 3-hydoxybutyrate, acetone, pyruvate, succinate, isobutyrate, and the majority of amino acids and decreased levels of ethanol, lipid, LDL and VLDL, demonstrated in the plasma of TACE group (Figure 2B).

According to Table 1, fifteen of the selected metabolites were sifted as characteristic metabolites for TACE subjects. Notably, an abnormally high lactate concentration (approximately 4-fold higher than control) was observed. Actually clinical reports from the medical staff show these subjects most suffer from advanced tumor stages. Therefore, their pathological states could be confirmed by enhanced glucose consumption and lactate production [29]. The amount of characteristic metabolites of TACE group was less than that of HCC group. Unfortunately, we failed to establish a reliable OPLS-DA model (Figure 2C) between HCC and TACE groups since this model exhibited a quite low Q^2^ value and no significant differences (*P* = 1). Possibly due to no statistically significant differences as shown in the PCA scores plots (Figure S1A). So it is suggestive that metabolic differences between control and TACE groups are more significant than that between HCC and TACE, along with more common metabolic features but little differences. However, when we compare the effects of different treatments, we observed a big difference.

**Figure 3:**
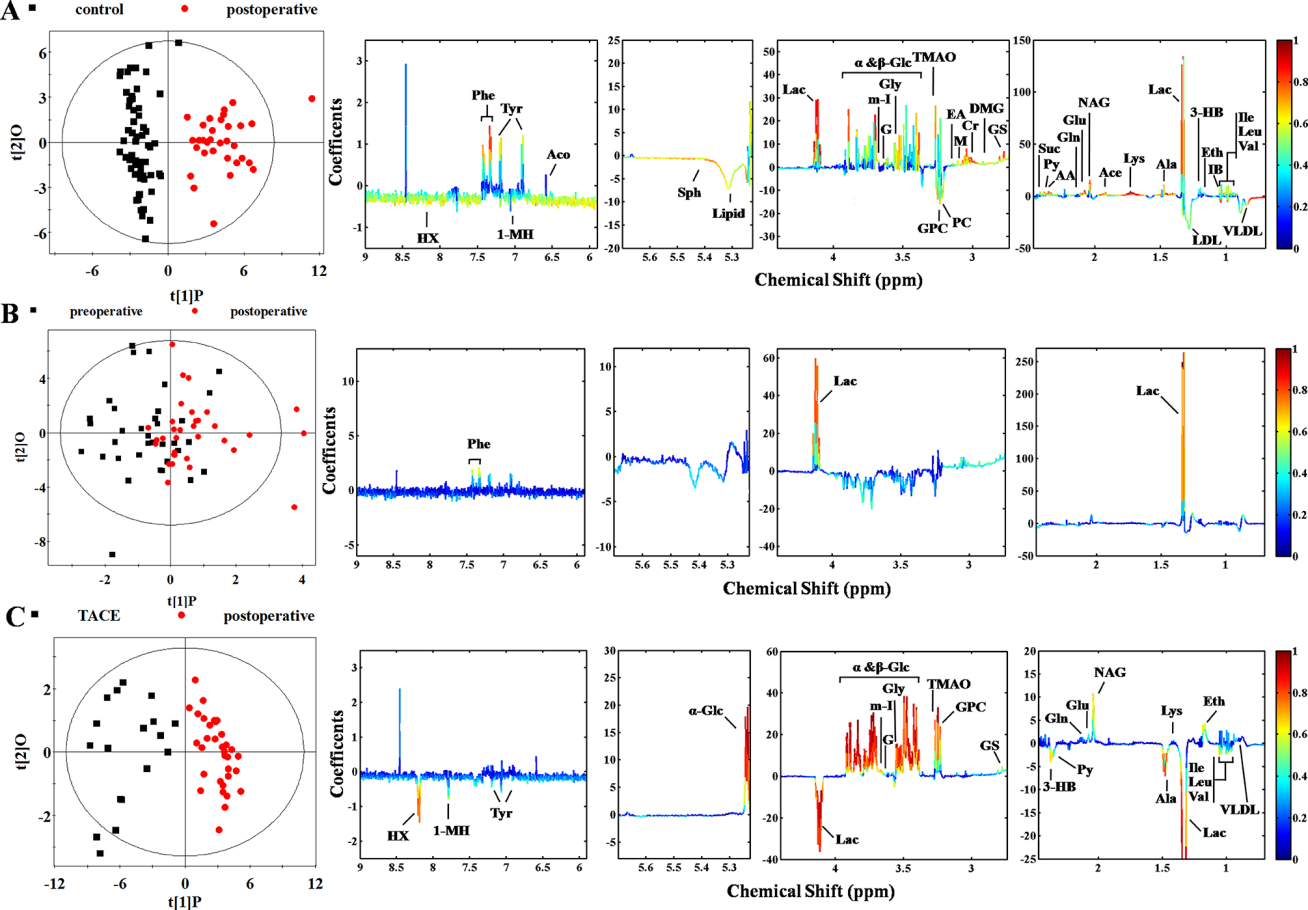
Score and loading plots for determination of metabolites responsible for plasma from groups of control, surgery, and TACE via OPLS-DA analyses (**A**) Control versus post-operative group (R^2^X = 48.2%, R^2^Y = 0.841, Q^2^ = 0.792, *P* < 0.0001); (**B**) pre- versus post-operative group (R^2^X = 29.3%, R^2^Y = 0.298, Q^2^ = −0.150, *P* = 1); (**C**) TACE versus post-operative group (R^2^X = 45.7%, R^2^Y = 0.837, Q^2^ = 0.713, *P* < 0.0001).

The surgical treatment did not significantly improve the metabolic profiles of HCC patients (Figure S1B and Figure 3A). According to the OPLS-DA model, many discriminatory metabolites were identified from post-operative group (Figure 3A). Even after the confirmation via *t*-test (Table 1), there are still 27 metabolites left as characteristic metabolites. Such result indicated that plasma metabolome still demonstrated increased glucose processing through glycolysis to pyruvate and other intermediates in TCA cycle, as well as lipid biosynthesis and glutaminolysis at post-surgery [30]. The similar result was also get from comparison of pre- and post-operative subjects, where mixed differentiation demonstrated no essential difference (Figure S1C and Figure 3B). Although lactate and phenylalanine are identified, further *t*-test eliminates the latter (Table 1). It is puzzling because the surgery is supposed to relieve the activity of cancer cells. A possible cause is the time point of blood sampling, since the majority of post-operative blood samples were collected in 24 h after surgery. It is obvious that only one day is deficient to suppress tumor activity.

Analysis of TACE and post-operative subjects was also undertaken to investigate treatment-induced metabolic alteration (Figure S1D and Figure 3C). Significant differentiation was achieved. A series of discriminating metabolites were identified (right panel of Figure 3C) and some of them were confirmed by *t*-test screening (Table 1). Notably, increased production of lactate and pyruvate were observed in both TACE and post-surgery plasma comparing with control, possibly due to declining pyruvate into the citric acid cycle in mitochondria [27]. Actually, different levels of glucose consumption reflect the activity of tumor, suggesting that recurrence or metastasis of HCC shows a stronger vitality.

### Metabolic pathways analysis

**Figure 4:**
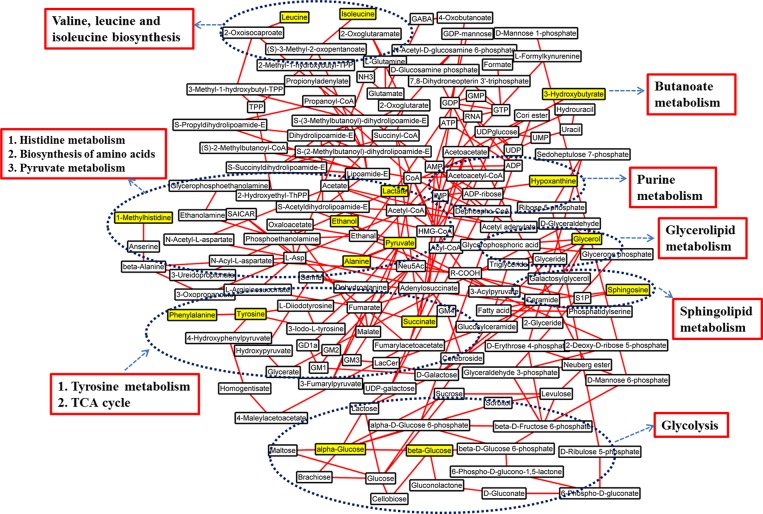
Schematic diagram of human HCC metabolic pathways The metabolites in yellow backgrounds are the characteristic metabolites of HCC patients without surgery or TACE. The corresponding metabolic pathways are demonstrated in the dark blue ellipse and the names are identified by red box.

A MATLAB-based tool was used to draw the map of relative biochemical pathways [31], and the custom sub-networks for HCC were created by using main substrate-product pairs as defined by KEGG online database. In the HCC-related metabolic network (Figure 4), eleven biochemical pathways, including energy metabolism like glycolysis, biosynthesis of amino acids, metabolisms of amino acids, pyruvate, glycerolipid, sphingolipid, purine and butanoate, and TCA cycle, were involved in pathological changes of HCC patients.

**Figure 5:**
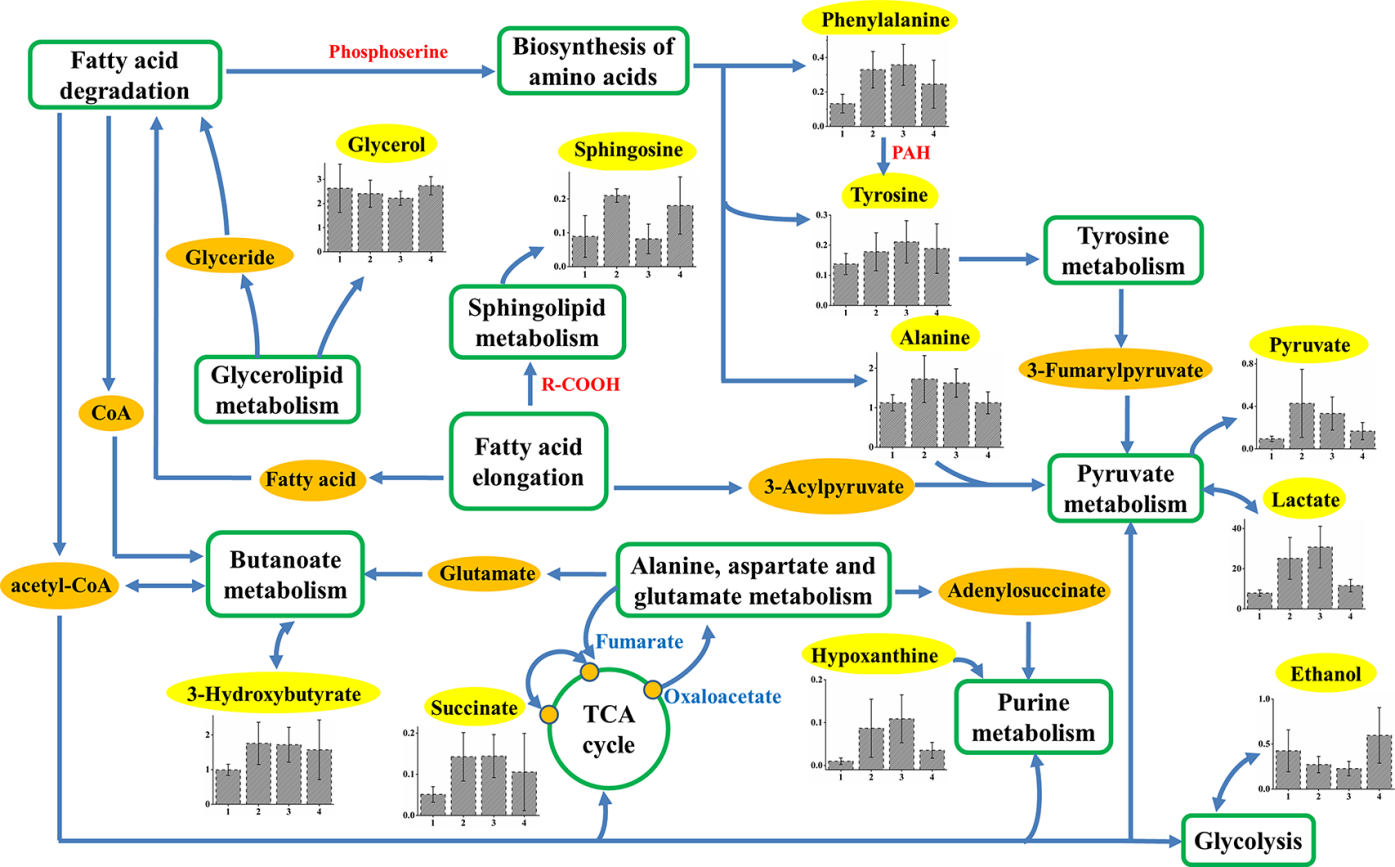
Schematic diagram of human HCC metabolic pathways that are not significant response to TACE or surgical treatments Relative levels of characteristic compounds within these pathways are also shown. 1, 2, 3, and 4 stand for plasma samples taken from control, HCC only, TACE, and surgery groups, respectively. The metabolites in yellow backgrounds are the common characteristic metabolites in HCC-related plasma samples, and the corresponding metabolic pathways are demonstrated in the green box or circle.

Furthermore, by extracting the common characteristic metabolites from HCC only, TACE, and surgery groups, the involved metabolic pathways composed the insensitive response to clinical treatments (Figure 5). In general, there are 12 common characteristic metabolites (lipid is not shown here) and 11 corresponding pathways are involved, which indicate insensitivity after TACE or surgical treatment. Increased levels of 3-hydroxybutyrate involved in butanoate metabolism, indicate upregulation of the synthesis of ketone bodies. This results in the accumulation of acetyl-CoA and sequentially alters the TCA cycle, and purine, pyruvate metabolisms [32, 33]. Elevated alanine, phenylalanine, and tyrosine levels in HCC and TACE groups indicate that abnormal biosynthesis of amino acid metabolism still occurs even after clinical treatments. Others found these aromatic amino acids to be elevated in HCC patients with liver failure [19, 34], and their increases were attributed to high levels of biosynthesis precursors [35, 36]. Therefore, TACE or surgical treatment may fail to inhibit perturbation involved in tyrosine and phenylalanine metabolisms. In addition, lactate, which is also involved in pyruvate metabolism, is elevated in all HCC groups with different degrees (Figure 5). This finding represents a major difference in the interconversion of lactate and pyruvate. According to these features, pyruvate metabolism of these patients may have been disrupted in some way, but the treatments did not improve the disorders. This metabolic network suggested that these biochemical pathways should be targeted for the therapeutic purpose of HCC patients in clinical management.

### Classification of HCC patients by plasma-derived characteristic metabolites

**Figure 6:**
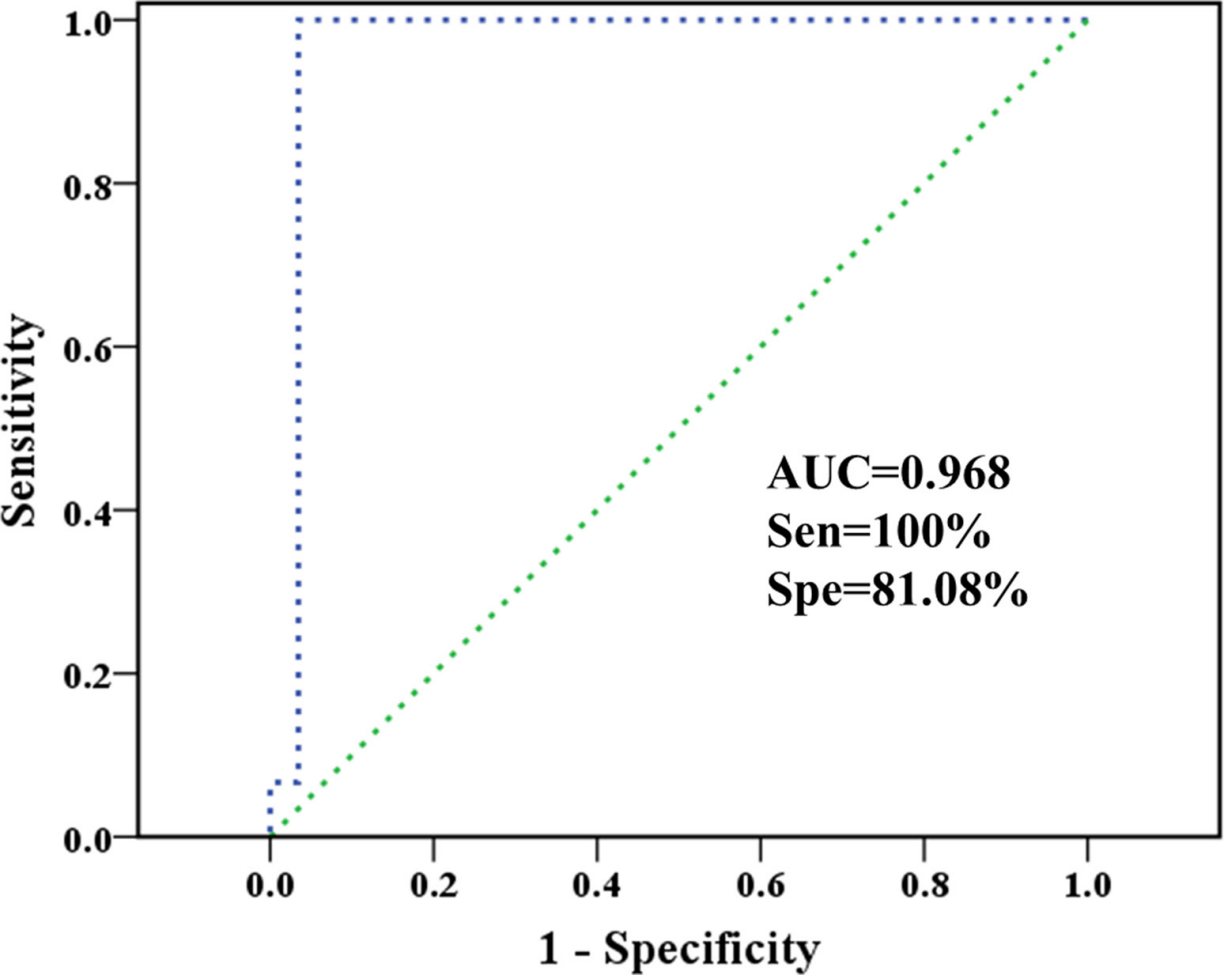
Classification of healthy controls and HCC patients based on plasma-derived characteristic metabolites The ROC curve of SVC classifier, as well as the sensitivity, specificity, AUC, and diagnostic reference line are shown.

The characteristic metabolites were fed back to explore the ability in classifying HCC and control subjects combining with support vector machines (SVM) via LIBSVM package. We randomly extracted 50% samples from each group as training sets and remainders were used as validation sets. The receiver operating characteristic (ROC) curve of support vector classification (SVC)-based prediction is shown in Figure 6, along with the overall performances including area under the curve (AUC) and the corresponding diagnostic sensitivity and specificity.

The AUC for a perfect case will be 1.000. No control sample was misclassified, making the sensitivity reach 100% while specificity is only 81.08%. Besides, these results showed that SVC algorithm based on characteristic metabolites data sets performed robustly and again HCC patients have different metabolic profiles accountable for their biological properties. Moreover, the potential and extend of transforming characteristic metabolites to disease biomarkers still need to be tested in future clinical applications.

## MATERIALS AND METHODS

### Ethics statement and clinical subjects

This study was approved by the Ethics committee of Xiamen University. The human plasma samples were used in accordance with the guidelines of Zhongshan Hospital Xiamen University. We recruited 57 patients with HCC (33 patients underwent surgical treatment), 18 patients underwent TACE (for recurrence or metastasis of HCC), and 60 healthy control subjects at the department of hepatobiliary surgery from April 2012 to May 2014. Following informed consent obtained from each subject, patients needed to fulfill the following inclusion criteria: (1) biopsy-proven HCC; (2) no infection by the human immunodeficiency hepatitis C viruses. HCC patients were diagnosed according to histological evidence or the combination of imaging techniques that showed this morphologic aspect plus an AFP level of 400 ng/mL or more. And the clinical information such as age, gender, and some important biochemical indexes for HCC patients and healthy subjects was presented in Table 2. The blood AFP, bilirubin and three enzyme levels correlating to liver function were measured on all subjects. The AFP levels were significantly higher in HCC than in control. Besides, associated HBV antibody was also noted in HCC patients.

**Table 2:** Clinical information for healthy humans and HCC patients

Parameters	Control	HCC	TACE	Pre-surgery	Post-surgery
Number	60	24	18	33	33
Age	58.5 ± 11.0	57.3 ± 3.3	60.5 ± 8.7	50.1 ± 4.2	50.1 ± .2
Male/Female	36/24	20/4	15/3	27/6	27/6
AFP value (ng/mL)	22 ± 1.1	463.2 ± 199.8	55387.5 ± 5399.5	62105.4 ± 2840.6	15865.3 ± 1103.9
HBsAg (positive %)	-	100	89	50	50
ALP (IU/L)	72.0 ± 18.3	154.3 ± 20.3	124.8 ± 18.4	152.5 ± 22.3	129.8 ± 17.4
ALT (IU/L)	18.0 ± 6.4	73.6 ± 11.4	97.1 ± 23.8	120.0 ± 23.1	56.5 ± 9.7
AST (IU/L)	22.0 ± 10.4	74.5 ± 13.3	154.2 ± 54.0	151.2 ± 58.7	81.9 ± 13.2
D-BIL (μmol/L)	2.5 ± 1.2	7.1 ± 1.2	11.0 ± 4.4	12.9 ± 4.7	5.9 ± 0.8
T-BIL (μmol/L)	9.2 ± 3.2	21.6 ± 4.9	14.6 ± 2.4	22.5 ± 5.3	14.3 ± 2.2

Data are presented as mean ± SE.

Abbreviations: AFP, alpha-fetoprotein; HBsAg, hepatitis B surface antigen; ALP, alkaline phosphatase; ALT, alanine aminotransferase; AST, aspartate transaminase; D-BIL, direct bilirubin; T-BIL, total bilirubin.

### Plasma sample collection and preparation

Venous blood samples were preprandially collected in the morning during routine clinical blood work using heparin tubes or citrate-rinsed tubes. During this procedure, blood samples from HCC patients underwent surgical treatment were collected before and after surgical treatment, respectively. Plasma was separated by centrifugation at 1,000 g at 4°C for 10 min and then immediately stored at −80°C until further analyses. Before NMR analyses, the plasma samples were thawed at room temperature, and 400 μL of aliquots were mixed with 200 μL of D_2_O-prepared phosphate-buffered saline (PBS, pH = 7.4) and centrifuged at 10,000 g for 10 min at 4°C. Subsequently, 550 μL of the supernatant was transferred to a 5 mm NMR tube, and NMR acquisition was immediately performed.

### ^1^H NMR spectroscopy

The ^1^H NMR measurements of plasma samples were performed using a 500 MHz Varian NMR spectrometer equipped with a triple resonance probe, operating at a ^1^H frequency of 499.74 MHz. The experimental temperature was set to 293 K and the 90° pulse length was calibrated individually for each sample. Standard 1D ^1^H spectra were acquired with a Carr-Purcell-Meiboom-Gill (CPMG) spin-echo pulse sequence with relaxation time of 2 s and acquisition time of 1 s. A total of 128 scans with a spectral width of 10,000 Hz were collected into 64 K data points for all NMR experiments.

### Data preprocessing and pattern recognition

All free induction decays (FIDs) were multiplied by a 1.0 Hz exponential line broadening factor prior to Fourier Transformation. The collected NMR spectra were manually phased and baseline corrected using the software MestReNova (version: 8.1.2, Mestrelab Research S.L., Spain) and referenced to the CH_3_ resonance of lactate at δ1.33. The peaks of metabolites observed in the plasma ^1^H NMR spectra were assigned with reference to published data [36, 37] and confirmed by HMDB database [38]. The regions of δ2.47–2.74, δ4.40–5.30, andδ5.70–5.90 were excluded to remove the effects of citrate, urea and variation in residual water. Subsequently, the spectra were divided into regions of 0.002 ppm and integrated in the region of 0.50–9.00 ppm. To account for variations in sample concentration, the spectra were normalized to the total sum of the spectrum before multivariate statistical analysis.

Protocols for statistical analysis have been described previously in full by Li *et al.* [39]. Briefly, PCA and OPLS-DA were carried out using SIMCA-P + v14.0 (Umetrics, Sweden). Data used in PCA were mean-centered scaled while in OPLS-DA were Pareto scaled. The optimal number of orthogonal components for building OPLS-DA models was selected using a 15-fold cross validation procedure. The goodness of fit and prediction parameters of OPLS-DA models, R^2^ and Q^2^ were calculated, and the corresponding probability (*P*-value) of significant difference between the pair-wise groups were obtained by CV-ANOVA. Visualization of results was based on score plots of the first two components, and a correlation coefficient of |r| > 0.45 was used as the cut-off value for determination of characteristic metabolites with significance level of *P* < 0.05 according to Student's *t*-test analysis.

Finally, the associative characteristic metabolites were fed back to identify HCC and control subjects by combining with SVM via LIBSVM package (Machine Learning and Data Mining Group) [40]. Since only about a dozen of metabolites were involved, SVM algorithm proposed by Vapnik was suitable for such small-sample problems [41]. The task of LIBSVM was SVC algorithm. Such algorithm aims to construct optimal hyper-plane in a higher dimensional space that maximal margin two classes according to the input data sets. The type is C_SVC with kernel function of the radial basis function. Automatic optimization parameters together with leave-one-out cross-validation were utilized to predict unknown samples and evaluate the reliability of SVC models.

## CONCLUSIONS

In this study, NMR-based metabolomic techniques were used to identify the characteristic metabolic profiles of plasma from HCC patients in different pathological backgrounds. High consumption of glucose was revealed in HCC patients along with large amount of intermediates and end-products from aerobic glycolysis. Most of the characteristic metabolites were consistent with previous studies except the addition of some novel discoveries. A series of biological behaviors caused by high glucose consumption have become a common feature in HCC patients. Patients underwent TACE seem to be more discriminatory from control than other HCC patients, which is consistent with that TACE treatment was targeted at advanced subjects in clinical practice. The key response of surgery was revealed by an overall increase in energy metabolism. However, the TACE and surgical treatments didn't immediately induce obvious improvement in metabolic profiles. The corresponding metabolic pathway analysis vastly extends clinical relevance and effects of our proposed biomarkers. We are also aware that these results should be validated by a larger cohort of samples, and that external validations are essential to test the clinical validity of characteristic metabolites-derived classification models. The short interval of blood sampling after surgery is likely to expose one of the limitations of this study, and the confirmation should be contained in further work.

## SUPPLEMENTARY MATERIALS


