# Supplementary material for: Plasma metabolomic analysis of human hepatocellular carcinoma: Diagnostic and therapeutic study
Source: Oncotarget. 2016 Jun 17;7(30):47332–42. doi: 10.18632/oncotarget.10119 (PMC5216945; doi:10.18632/oncotarget.10119)
Supplement: Supplementary file 1 [file oncotarget-07-47332-s001.pdf]

# Plasma metabolomic analysis of human hepatocellular carcinoma: Diagnostic and therapeutic study

## Supplementary Materials

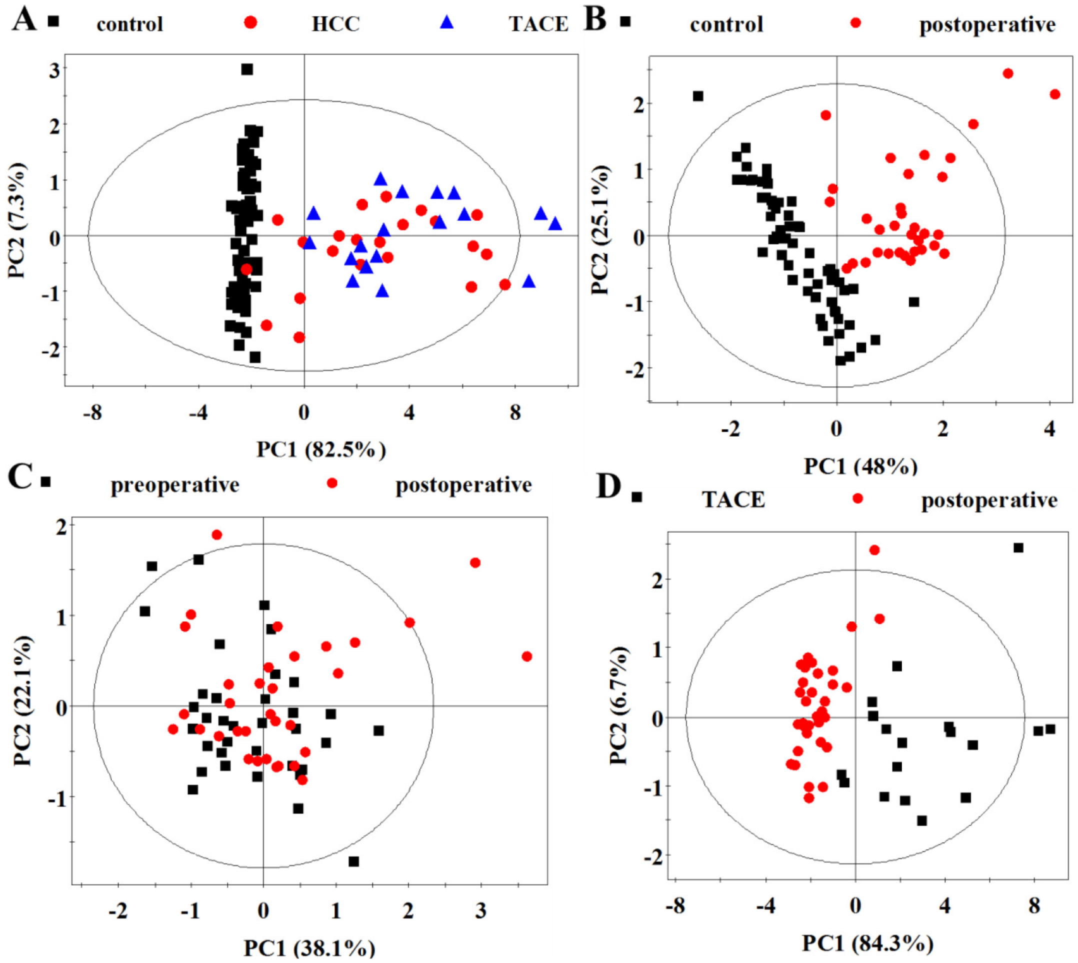


**Supplementary Figure S1: Scores plots of the first two components in PCA analysis for plasma samples.** (**A**) Control versus HCC and TACE groups ($R^2X$ = 89.8%, $Q^2$ = 0.885); (**B**) control versus postoperative group ($R^2X$ = 73.1%, $Q^2$ = 0.677); (**C**) pre- versus post-operative group ($R^2X$ = 60.2%, $Q^2$ = 0.512); (**D**) TACE versus postoperative group ($R^2X$ = 91%, $Q^2$ = 0.888).

## Supplementary Table S1: Assignments of metabolites from $^1$H NMR analysis of plasma

| metabolite | Abbr. | $^1$H Shift (multiplicity) |
|---|---|---|
| low-density lipoprotein | LDL | 0.85 (br[a]), 1.28 (br) |
| very low-density lipoprotein | VLDL | 0.88 (br), 1.30 (br), 1.58 (br) |
| isoleucine | Ile | 0.94 (t), 1.01 (d) |
| leucine | Leu | 0.96 (t) |
| valine | Val | 0.99 (d), 1.04 (d) |
| isobutyrate | IB | 1.07 (d) |
| ethanol | Eth | 1.19 (t) |
| 3-hydroxybutyrate | 3-HB | 1.20 (d), 2.31 (dd), 2.41 (dd), 4.16 (m) |
| lactate | Lac | 1.33 (d), 4.11 (q) |
| lysine | Lys | 1.46 (m), 1.73 (m), 1.91 (m), 3.03 (t), 3.76 (t) |
| alanine | Ala | 1.48 (d) |
| acetate | Ace | 1.92 (s) |
| lipid | L | 2.02 (br), 2.24 (br), 2.78 (br), 5.31 (br) |
| N-acetylglycoprotein | NAG | 2.04 (s) |
| glutamate | Glu | 2.08 (m), 2.12 (m), 2.35 (m), 3.78 (t) |
| glutamine | Gln | 2.14 (m), 2.45 (m), 3.78 (t) |
| methionine | Met | 2.14 (s) |
| acetone | Act | 2.23 (s) |
| acetoacetate | AA | 2.28 (s) |
| pyruvate | Py | 2.37 (s) |
| succinate | Suc | 2.41 (s) |
| guanidinosuccinate | GS | 2.80 (dd), 4.25 (m) |
| N,N-dimethylglycine | DMG | 2.93 (s) |
| creatine | Cr | 3.04 (s), 3.93 (s) |
| malonate | M | 3.11 (s) |
| ethanolamine | EA | 3.15 (t) |
| choline | Cho | 3.20 (s) |
| phosphocholine | PC | 3.21 (s) |
| glycerophosphocholine | GPC | 3.23 (s) |
| β-glucose | β-Glc | 3.26 (dd), 3.41 (t), 3.46 (dd), 3.49 (t), 3.73 (dd), 3.90 (dd) |
| trimethylamine N-oxide | TMAO | 3.27 (s) |
| methanol | Mol | 3.36 (s) |
| α-glucose | α-Glc | 3.42 (t), 3.54 (dd), 3.72 (t), 3.84 (m), 5.24 (d) |
| glycine | Gly | 3.56 (s) |
| glycerol | G | 3.57 (m), 3.66 (dd), 3.79 (m) |
| *myo*-inositol | m-I | 3.61 (m), 4.05 (m) |
| sphingosine | Sph | 5.42 (m), 5.70 (m) |
| *cis*-aconitate | Aco | 6.59 (s) |
| tyrosine | Tyr | 6.90 (d), 7.19 (d) |
| 1-methylhistidine | 1-MH | 7.06 (s), 7.79 (s) |
| phenylalanine | Phe | 7.33 (d), 7.37 (m), 7.42 (m) |
| hypoxanthine | HX | 8.19 (s), 8.21 (s) |
| formate | For | 8.46 (s) |

[a]Multiplicity: br broad resonance, t triplet, d doublet, dd doublet of doublets, m multiplet, q quartet, s singlet.

**Supplementary Table S2: Summary of *P*-value during *t*-test of characteristic metabolites from different pathological backgrounds**

| Metabolite | Control versus HCC | Control versus TACE | HCC versus TACE | Control versus post-surgery | Pre-surgery versus post-surgery | TACE versus post-surgery |
|---|---|---|---|---|---|---|
| low-density lipoprotein | 0.0139* | 0.0410 | 0.9617 | 0.0063 | 0.1357 | 0.9408 |
| very low-density lipoprotein | 0.0000 | 0.0014 | 0.4184 | 0.0000 | 0.1484 | 0.6822 |
| Isoleucine | 0.0004 | 0.4588 | 0.0742 | 0.4134 | 0.3322 | 0.8780 |
| Leucine | 0.0046 | 0.1285 | 0.2145 | 0.8299 | 0.0415 | 0.3382 |
| Isobutyrate | 0.0001 | 0.7953 | 0.0241 | 0.0010 | 0.8581 | 0.0472 |
| Ethanol | 0.0057 | 0.0028 | 0.2981 | 0.0282 | 0.0761 | 0.0000 |
| 3-Hydroxybutyrate | 0.0000 | 0.0000 | 0.6185 | 0.0000 | 0.5466 | 0.2947 |
| Lactate | 0.0000 | 0.0000 | 0.8390 | 0.0004 | 0.0178 | 0.0000 |
| Alanine | 0.0000 | 0.0147 | 0.0127 | 0.0230 | 0.2058 | 0.5064 |
| Acetate | 0.2959 | 0.8246 | 0.2964 | 0.0117 | 0.9767 | 0.1455 |
| Lipid | 0.0004 | 0.0107 | 0.2845 | 0.0038 | 0.5113 | 0.7166 |
| Glutamate | 0.1948 | 0.7261 | 0.1989 | 0.0364 | 0.0720 | 0.2467 |
| Acetone | 0.0099 | 0.0000 | 0.1583 | 0.0088 | 0.7290 | 0.5496 |
| Acetoacetate | 0.3206 | 0.6369 | 0.7138 | 0.0001 | 0.9682 | 0.0376 |
| Pyruvate | 0.0000 | 0.0000 | 0.0018 | 0.0000 | 0.1752 | 0.0000 |
| Succinate | 0.0000 | 0.0000 | 0.3985 | 0.0000 | 0.9132 | 0.2050 |
| Guanidinosuccinate | 0.0205 | 0.1229 | 0.6242 | 0.0000 | 0.8794 | 0.0014 |
| N,N-Dimethylglycine | 0.4349 | 0.9933 | 0.1417 | 0.0000 | 0.3641 | 0.0002 |
| Creatine | 0.3904 | 0.5348 | 0.8934 | 0.9209 | 0.0396 | 0.5985 |
| Malonate | 0.3479 | 0.2186 | 0.6910 | 0.0000 | 0.0183 | 0.0000 |
| Ethanolamine | 0.5662 | 0.5778 | 0.9695 | 0.0000 | 0.1890 | 0.0016 |
| Phosphocholine | 0.1054 | 0.1430 | 0.9868 | 0.0293 | 0.6067 | 0.7669 |
| β-Glucose | 0.0055 | 0.1858 | 0.2707 | 0.0103 | 0.2946 | 0.0007 |
| Trimethylamine N-oxide | 0.2143 | 0.1760 | 0.9760 | 0.0423 | 0.9299 | 0.6394 |
| Methanol | 0.0000 | 0.0028 | 0.0010 | 0.0002 | 0.2240 | 0.5518 |
| α-Glucose | 0.0234 | 0.4233 | 0.2715 | 0.0018 | 0.0874 | 0.0003 |
| Glycerol | 0.0253 | 0.0069 | 0.3732 | 0.0008 | 0.0855 | 0.7178 |
| Sphingosine | 0.0482 | 0.0058 | 0.0370 | 0.0359 | 0.1236 | 0.0188 |
| *cis*-Aconitate | 0.0892 | 0.0116 | 0.5700 | 0.0000 | 0.3673 | 0.0357 |
| Tyrosine | 0.0022 | 0.0001 | 0.4785 | 0.0000 | 0.6619 | 0.6940 |
| 1-Methylhistidine | 0.0011 | 0.3740 | 0.0279 | 0.7990 | 0.2999 | 0.5466 |
| Phenylalanine | 0.0000 | 0.0016 | 0.8288 | 0.0000 | 0.9402 | 0.5097 |
| Hypoxanthine | 0.0000 | 0.0000 | 0.3926 | 0.0007 | 0.3600 | 0.0329 |
| Formate | 0.3144 | 0.3339 | 0.7726 | 0.0000 | 0.6780 | 0.0000 |

*$P < 0.05$ is considered significantly different.
